# Point-of-care Ultrasound Diagnosis of Pulmonary Hydatid Cyst Disease Causing Shock: A Case Report

**DOI:** 10.5811/cpcem.2021.5.52264

**Published:** 2021-08-27

**Authors:** Alexandra Hill, Marco Guillén, David Martin, Andrea Dreyfuss

**Affiliations:** * Highland Hospital - Alameda Health System, Department of Emergency Medicine, Oakland, California; † Hospital Nacional Adolfo Guevara Velasco, Department of Emergency Medicine, Cusco, Peru

**Keywords:** Point-of-care ultrasound, POCUS, shock, dyspnea, hydatid cyst, case report

## Abstract

**Introduction:**

Point-of-care ultrasound (POCUS) is accepted as an important tool for evaluating patients presenting to the emergency department (ED) with dyspnea^1^ and undifferentiated shock.^2^ Identifying the etiology and type of shock is time-critical since treatments vary based on this information. Clinicians typically rely on the history, exam, and diagnostics tests to identify the etiology of shock. In resource-limited settings where there is reduced access to timely laboratory and diagnostic studies. The use of POCUS enables rapid classification and directed treatment of shock. Additionally, POCUS can aid in the diagnosis of rarer tropical diseases that can be important causes of shock in resource-limited settings.

**Case Report:**

We discuss a case of a pediatric patient who presented to an ED in Cusco, Peru, with acute dyspnea and shock. Point-of-care ultrasound was used to expedite the diagnosis of a ruptured pulmonary hydatid cyst, guide proper management of septic and anaphylactic shock, and expedite definitive surgical intervention.

**Conclusion:**

In resource-limited settings where there is reduced access to timely laboratory and diagnostic studies, the use of POCUS enables rapid classification and directed treatment of shock.

## INTRODUCTION

Hydatid cysts caused by infection with *Echinococcus granulosus* are recognized as a neglected tropical disease causing significant morbidity worldwide.^3^ Humans and other intermediate hosts such as sheep are infected after ingesting eggs in the feces of the definitive host, dogs.^4^ Patients from endemic regions, including South America, the Middle East, China, Australia, and Africa, are at risk for hydatid cysts.^5^ There have also been cases of local infection reported in the southwest United States.^4^ Adults most commonly develop hydatid cysts in the liver (75%) and the lungs (15%).^4^ By contrast, children primarily develop pulmonary hydatid infections possibly because cysts grow best in compressible organs.^5^,^6^

Pulmonary hydatid cysts may present with symptoms related to mass effect, vascular communication, and rupture.^5^,^7^ Patients with ruptured pulmonary hydatid cysts may develop anaphylaxis, pulmonary embolism, pleural effusions, or superimposed bacterial infection.^5^,^7^–^10^ Ultrasound is the cornerstone of diagnosis of hydatid cysts in the liver^4^,^11^ and is favored over computed tomography (CT) due to its superior ability to identify “membranes, septae, and hydatid sand” within the abdomen.^6^ By contrast, the diagnosis of pulmonary hydatid cysts is more challenging and traditionally relies on a combination of chest radiograph (CXR), CT, serology, percutaneous aspiration, bronchoscopy, and/or surgical pathology.^7^,^12^ Ultrasound has been thought to be of limited utility due acoustic shadowing from the air-filled lungs.^12^,^13^

Point-of-care ultrasound (POCUS) is known to be useful in evaluating critically ill patients presenting with acute dyspnea^1^ and shock^2^ in the emergency department (ED). It can aid in the diagnosis and management of multiple etiologies of acute dyspnea, including pneumonia, heart failure, pleural effusion, pulmonary embolism, pericardial effusion, and pneumothorax.^1^,^14^ Similarly, POCUS has been shown to be helpful in differentiating types of shock, especially obstructive, cardiogenic, and hypovolemic, in resource-limited settings.^2^ Here, we describe a case of a five-year-old male who presented to an ED in Cusco, Peru, with acute dyspnea and shock. We demonstrate how the use of POCUS led to the unusual diagnosis of a ruptured hydatid cyst and helped to guide proper management of the patient’s respiratory failure and shock.

## CASE REPORT

A five-year-old previously healthy male was brought to the ED in Cusco, Peru, by his parents, who reported two days of fever, cough, rhinorrhea, pruritus, and decreased appetite. His temperature was 38.3° Celsius, heart rate was 150 beats per minute, respiratory rate was 52 breaths per minute, oxygen saturation was 77% on room air, and blood pressure was 100/60 millimeters of mercury (mm Hg). He appeared lethargic, and his capillary refill was less than two seconds. He was using accessory respiratory muscles and had wheezing and diminished vesicular breath sounds in the left hemithorax.

**Image 1:**
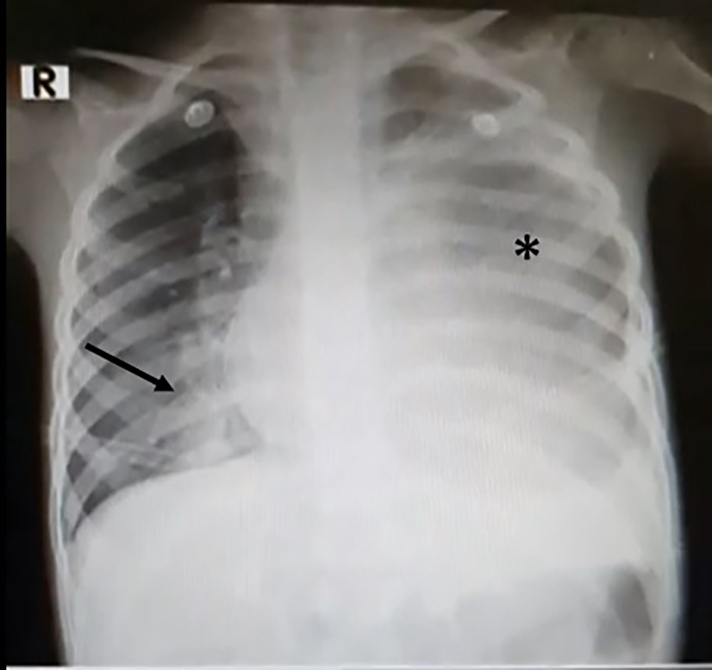
Chest radiograph of five-year-old patient obtained in the emergency department. The left hemithorax is radiopaque, concerning for a possible large pleural effusion (asterisk). There is a normal cardiac silhouette and hazy interstitial opacities concerning for an infectious process present in the right hemithorax (arrow).

His labs were notable for white blood cell count of 4.35×10^3^ per cubic millimeter (mm^3^) (reference range: 5×10^3^ – 10×10^3^ / mm^3^) with 84.8% polymorphonuclear leukocytes, hemoglobin of 9.5 grams per deciliter (g/dL) (reference range: 14–17 g/dL), platelets of 474,000 per microliter (μL) (reference range: 140,000–500,000/μL), and a creatinine of 0.24 milligrams (mg)/dL (reference range: 0–0.5 mg/dL). His arterial blood gas demonstrated metabolic acidosis with hypoxemia. The CXR showed radiopacity of the entire left hemithorax consistent with a massive pleural effusion (Image 1).

Based on the patient’s overall presentation and CXR findings, the pediatrician suspected the patient was presenting with acute hypoxemic respiratory failure and septic shock from pneumonia complicated by a large parapneumonic effusion. Given the patient’s tachypnea, hypoxia, flash capillary refill, and severe tachycardia, there was concern for impending respiratory failure and warm shock. The pediatrician consulted the emergency physician to perform an ultrasound-guided diagnostic and therapeutic thoracentesis.

**Image 2:**
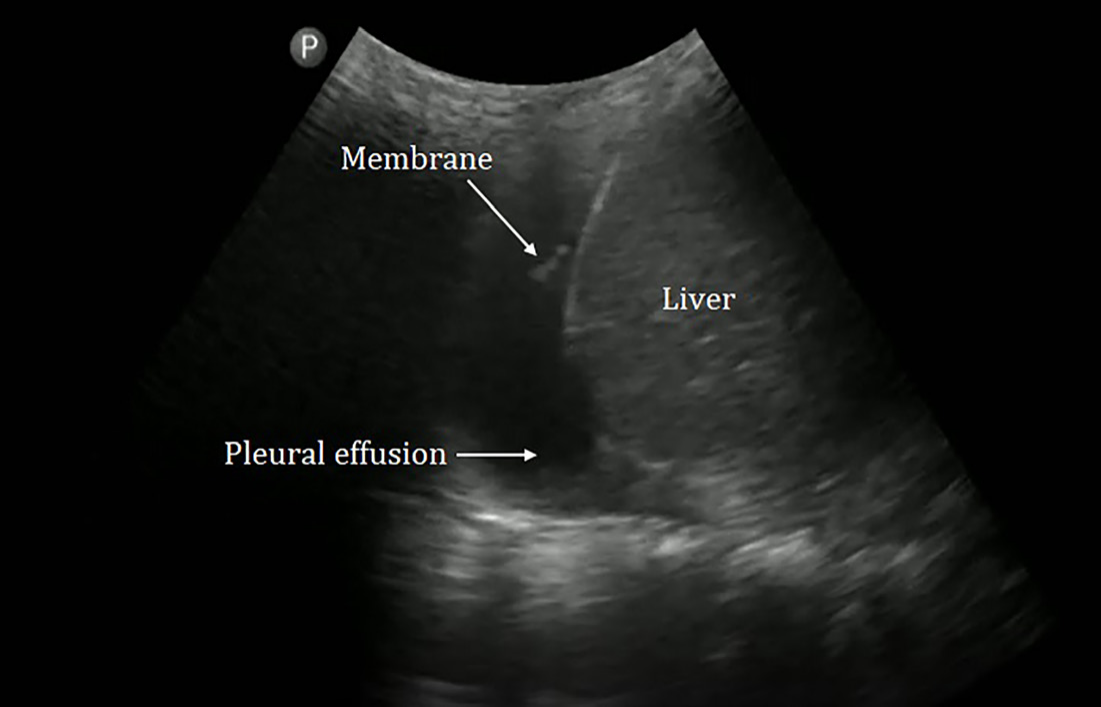
Point-of-care ultrasound of the right hemithorax showing a hyperechoic linear structure (suggestive of a membrane) floating in anechoic fluid (a pleural effusion).

The emergency physician began his POCUS examination by performing a “triple scan,” which includes cardiac, inferior vena cava, and pulmonary ultrasound windows to evaluate for potential causes of the patient’s acute dyspnea and shock.^1^ The POCUS revealed a preserved ejection fraction on the parasternal long view and a collapsing inferior vena cava on the subxiphoid view consistent with the pediatrician’s concern for septic shock. Point-of-care ultrasound of the right hemithorax revealed a pleural effusion, a hyperechoic structure suggestive of a membrane in the pleural cavity, multiple B-lines, and a subpleural consolidation (Image 2, Video 1).

**CPC-EM Capsule**

What do we already know about this clinical entity?

*Hydatid cyst disease can lead to complications including sepsis and anaphylaxis. Diagnosis of pulmonary involvement often requires a multimodal approach.*

What makes this presentation of disease reportable?

*Emergency providers used point-of-care ultrasound (POCUS) to diagnose and guide management of a pediatric patient with shock due to a ruptured pulmonary hydatid cyst.*

What is the major learning point?

*Point-of-care ultrasound can identify ruptured or multivesicular pulmonary hydatid cysts due to their unique ultrasonographic appearance.*

How might this improve emergency medicine practice?

*In undifferentiated shock, POCUS is an excellent tool for evaluating critically ill patients, including those who may be suffering from rare tropical diseases.*

**Image 3:**
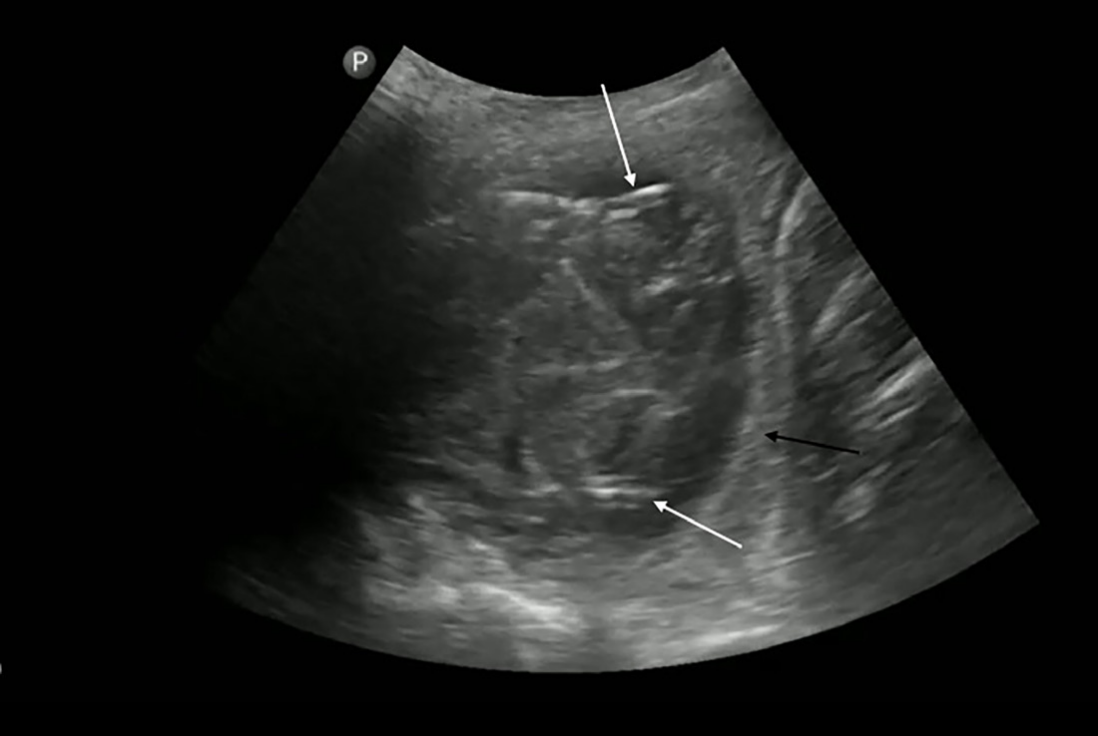
Point-of-care ultrasound of the left hemithorax showing the “honeycomb” appearance of a multivesicular cyst with double echogenic lines^6^ (white arrows) known as the “wall sign”^5^ and internal “serpentine” linear structures delineating the daughter cysts.^6^ This cyst occupies nearly the entire volume of the left hemithorax (black arrow indicates the diaphragm).

In the left hemithorax, a complex, double-layered structure was noted on POCUS (Image 3, Video 2). This was consistent with a “wall sign” and was thought to represent a cyst occupying the entire pleural cavity. This structure contained hypo- and hyperechoic regions (suggestive of multiple membranes), floating in heterogeneous fluid, which appeared to be pus.

Based on these ultrasound images and the endemic nature of *E granulosus* in Cusco, a ruptured hydatid cyst was suspected to be the culprit. Due to these unexpected findings, the emergency physician aborted the planned thoracentesis since disrupting the cysts further could prove fatal to the patient by releasing “highly antigenic fluid” into the pleural space.^10^ A chest CT corroborated the diagnosis of a ruptured hydatid cyst in the left lung. No abnormal CT findings were noted in the radiologist’s read of the right hemithorax, despite the abnormal findings seen on POCUS.

The patient was subsequently intubated for respiratory failure. He became hypotensive to 80/50 mm Hg and was started on an epinephrine infusion for treatment of septic and anaphylactic shock. Both were a concern since the patient had fever, cough, and evidence of purulent pleural fluid on POCUS, which suggested infection, in addition to wheezing, pruritus, and hypotension, which was concerning for anaphylaxis. The patient was also given hydrocortisone, vancomycin, ceftriaxone, meropenem and albendazole. Cardiothoracic surgery took him to the operating room for a thoracotomy where they removed a giant, multivesicular hydatid cyst containing purulent material that comprised the entire lower lobe and part of the upper lobe of the left bronchus. Their findings were consistent with superinfection of the hydatid cyst as well as rupture. Surgical pathology later confirmed the diagnosis: anhistic membranes of a hydatid cyst with active microorganisms.

Post-operatively, the patient improved gradually. On hospital day 12, he was transferred out of the intensive care unit, and on hospital day 25 he was discharged home with albendazole.

## DISCUSSION

This case highlights why POCUS is such a powerful diagnostic tool for the emergency physician, especially in resource-limited settings. Not only is POCUS a highly versatile and relatively low-cost technology, but it is also portable and readily available at the bedside of a critically ill ED patient, which can expedite care when there is lack of access to rapid CT in overburdened EDs or when the patient is too unstable for transportation. Our case also demonstrates how POCUS averted a potentially harmful procedure (thoracentesis) and guided fluid management, antibiotic and steroid initiation, vasopressor selection, and consultation.

Point-of-care ultrasound has been shown to be effective in helping emergency physicians differentiate between many etiologies of shock^2^ and dyspnea,^1^ and our case shows that it can even identify etiologies as unusual as a ruptured pulmonary hydatid cyst. As mentioned previously, the diagnosis of pulmonary hydatid cysts typically relies on a multimodal approach because the appearance of pulmonary hydatid cysts on CXR and CT is nonspecific with a few exceptions.^4^,^6^,^12^,^13^ Ultrasound has a limited ability to evaluate structures that are deep to air-filled structures, thereby limiting its use in evaluating univesicular and centrally located pulmonary hydatid cysts.^12^,^13^ However, for multivesicular cysts and cysts surrounded by a pleural effusion, such as in ruptured hydatid cysts, ultrasound can have a higher specificity than other imaging modalities due to the acoustic window provided by the fluid-filled daughter cysts or effusion, respectively, which makes it easier to see cystic walls.^13^ One study of nine cases of pulmonary hydatid cysts inside of large pleural effusions showed that the cysts were very well visualized on ultrasound but could not be seen on CT.^13^

Various sonographic findings should lead physicians to consider pulmonary hydatid cyst disease. Sometimes it is possible to see a “wall sign,” which is created by the cyst and the surrounding pericyst in univesicular cysts or by neighboring walls of the daughter cysts in multivesicular cysts.^5^,^13^ Multivesicular cysts have a characteristic “honeycomb” appearance due to the presence of daughter cysts.^6^ Broken daughter cysts may appear as “serpentine” linear structures.^6^ When a membrane detaches within a cyst, it can create a “water lily sign.”^4^,^6^ Additionally, hydatid sand falling to the most dependent part of the cyst appears as the “snowstorm sign.”^4^,^6^ Emergency physicians should familiarize themselves with these unique sonographic findings when caring for patients living in or traveling from regions with endemic *E granulosus* infection.

## CONCLUSION

Point-of-care ultrasound should be considered an essential instrument for the evaluation and management of critically ill patients presenting to the ED, particularly in resource-limited settings, as it can change management and expedite definitive care. Furthermore, POCUS can be an important tool for diagnosing less common etiologies of shock and dyspnea, such as pulmonary hydatid cysts that may be endemic in resource-limited settings. Larger studies should be performed to evaluate the sensitivity and specificity of sonographic signs mentioned above for the diagnosis of pulmonary hydatid cysts as the studies cited were small, a common weakness of studies on neglected tropical diseases.

## Supplementary Information

Video 1 Point-of-care ultrasound of the right hemithorax showing B-lines and a membrane suspended in a pleural effusion coming in and out of view with the patient’s respiratory cycle.

Video 2 Point-of-care ultrasound of the left hemithorax demonstrating a large multivesicular cyst and two areas where the “wall sign” 5 is apparent, demarcating the daughter cysts. As the ultrasound probe is fanned, it is possible to see the spine sign above the diaphragm due to the presence of a pleural effusion surrounding the cyst.
